# A Critical Assessment of the Need for Harmonization of the Legal Framework Concerning Abusive Informal Debt Collection Practices in the European Union: Is Harmonization Possible and How Can it Best Be Attained?

**DOI:** 10.1007/s10603-021-09495-z

**Published:** 2021-08-30

**Authors:** C.-G. Stănescu

**Affiliations:** Faculty of Law, University of Copenhagen, Njalsgade 76, Office: 6A-3-32, 2300 Copenhagen, Denmark

**Keywords:** Debt collection, Credit servicing, Consumer protection, Abusive practices, Nonperforming loans, Internal market, Harmonization

## Abstract

The loss of jobs and the decline in real incomes caused by the 2008 financial crisis and the COVID-19 pandemic have affected consumers’ ability to repay their debts. These have led to high ratios of non-performing loans (NPLs), which affect the stability of the financial industry and undermine economic recovery. The result has been a need for faster debt enforcement and a drastic increase in abusive informal debt collection practices (IDCPs). In the EU, the need to regulate and harmonize abusive IDCPs surfaced in 2018 in connection to the Proposal for a Directive on Credit Servicers, Credit Purchasers and the Recovery of Collateral (CSDP). The directive would enable banks to outsource the servicing of NPLs to a specialized debt collector, but it contained no protection rules against abusive IDCPs. In this article, the researcher critically assesses the need for harmonization of the legal framework concerning abusive IDCPs in the EU, mainly from the standpoint of the initial and current text of the CSDP. Where necessary, the researcher will refer to both historical and comparative law perspectives. The researcher focuses on the legal character of informal debt collection, its relation to financial services, and its potential sui generis character. After that, the researcher will address the arguments for and against establishing pan-EU sector-specific legislation dedicated to IDCPs. Next, the researcher discusses the constitutional authority of the EU to regulate abusive IDCPs. Finally, the researcher will examine the interaction of the CSDP with other consumer (financial) protection instruments to identify the best solution for harmonizing abusive IDCPs at the EU level. The researcher will juxtapose several dichotomies: general versus sector-specific, procedural versus substantive, minimum versus maximum harmonization, and hard versus soft regulation. In the conclusion, the researcher shall synthesize the core problems and suggest an approach.

The emergence of the credit card made personal credit more accessible by removing the need for collateral (Geisst, 2013) and caused several critical transformations of the financial business.

*First*, the expansion of credit provoked significant changes in both the shopping and payment patterns of customers. The credit card freed money from the constraints of time, enabled people to use money not yet earned or received (Geisst, 2013; Weatherford, 1997), and “lured” them into more spending (Deville, 2015; Levoie, 2012). These changes, coupled with the elimination of legal restrictions on interest charges (Graeber, 2014), exposed the consumer to the vagaries of future incomes, an increased risk of over-indebtedness (Weatherford, 1997), the loss of personal goods (including household goods), predatory loans, and abusive debt collection practices (Brown, 2019; Geisst, 2013).

*Second*, the expansion of credit put enormous pressure on banks to “create” the credited amounts. In combination with weak prudential rules concerning capital reserves (i.e., Basel II), the expansion of credit and defaults exposed financial institutions to the risk of collapse. This, in turn, deterred them from making other loans that contribute to economic growth (Geisst, 2013). As risk increases with the amount of defaulted (consumer) credit, financial institutions became eager and willing to discard as much bad debt as possible (Geisst, 2013; Levoie, 2012), usually by assigning it to professional debt buyers and debt collectors.

*Third,* the introduction of credit cards changed the business patterns of the companies that expanded credit or were affected by it. For a fee, the credit card company took over both the responsibility and the risk of judging the customer’s creditworthiness (Weatherford, 1997). At the same time, recovery was increasingly externalized (Deville, 2015; Rock, 1973).

This process of “financialization,” coupled with the expansion of financial neoliberalism (Graeber, 2014; Lavoie, 2012; Vellucci, 2021), led to the exponential growth and increasing importance of the debt collection industry (Deville, 2015; Graeber, 2014; Stănescu, 2015).

In the EU, the credit card took off under neoliberal policies praising free, unregulated markets (Geisst 1973; Brown, 2019; Vellucci, 2021). It advanced in stages, coupled with the fall of communism and the former communist Member States (MS) accession into the EU. In the early 1970s, when US consumer credit was at its peak, debtors could still go to prison in Britain for noncompliance with the court’s order to repay (Deville, 2015), while debt and default were associated with a powerful stigma (Rock, 1973). It also meant that strong-arm debt collection—including threats, public shaming, or hardship for the debtor and her close circle—was regarded as a proper tool (Deville, 2015; Rock, 1973).

In recent years, the loss of jobs and the decline in real incomes caused by the 2008 financial crisis or the COVID-19 pandemic have affected consumers’ ability to repay their debts (Brown, 2019; Deville, 2015; Feretti, 2016; Riefa, 2020). These caused high ratios of non-performing loans (NPLs), which affect the stability of the financial industry and undermine economic recovery (Boeddu et al., 2020; EC 2020; Macchiarelli et al., 2019). The result has been a need for faster extra-judicial enforcement and a drastic increase in abusive informal debt collection practices (IDCPs). For this article, abusive IDCPs encompass all methods of private enforcement employed for debt recovery that (a) do not involve the judiciary or other state agents and (b) threaten consumers’ physical, psychological, or economic well-being.

In its international Guidance on Good Practices for Financial Consumer Protection, the World Bank (2017, p. 45) noted that abusive and aggressive debt collection “is an issue in many credit markets,” “often used by a range of financial service providers,” and requires “sound regulation.” In addition to the dangers posed to consumers, weak safeguards against abusive debt collection negatively affect the financial industry. They strengthen the calls for more cumbersome recovery processes, cause moratoriums on collection, and help debtors earn the sympathy of courts, leading to lengthy and expensive procedures and thus increasing the cost of financing. From this perspective, all stakeholders involved in debt collection—regulators, creditors, debt collectors, and consumers—should be equally interested in implementing an adequate regulatory regime, as confirmed by pan-EU empirical studies (Stănescu, 2021).

In 2020, the World Bank restated its concern about consumer exposure to abusive debt collection, emphasizing that “enhanced monitoring of aggressive and unscrupulous debt collection activities is crucial” during times of crisis and economic recession, such as that caused by the COVID-19 pandemic (Boeddu et al., 2020).

Regulating abusive IDCPs has not been a subject of specific concern in the EU and was left to the MS. Even so, IDCPs remain widely unregulated at the MS level as well. According to a survey conducted in 2020, two-thirds of EU consumer-debtors (281.47 million people, Fig. 1, red states) lack adequate protections due to the absence of national sector-specific legislation. Meanwhile, EU-wide regulation (i.e., the Unfair Commercial Practices Directive, or UCPD) plays a marginal role (Stănescu, 2020, 2021).

**Fig. 1:**
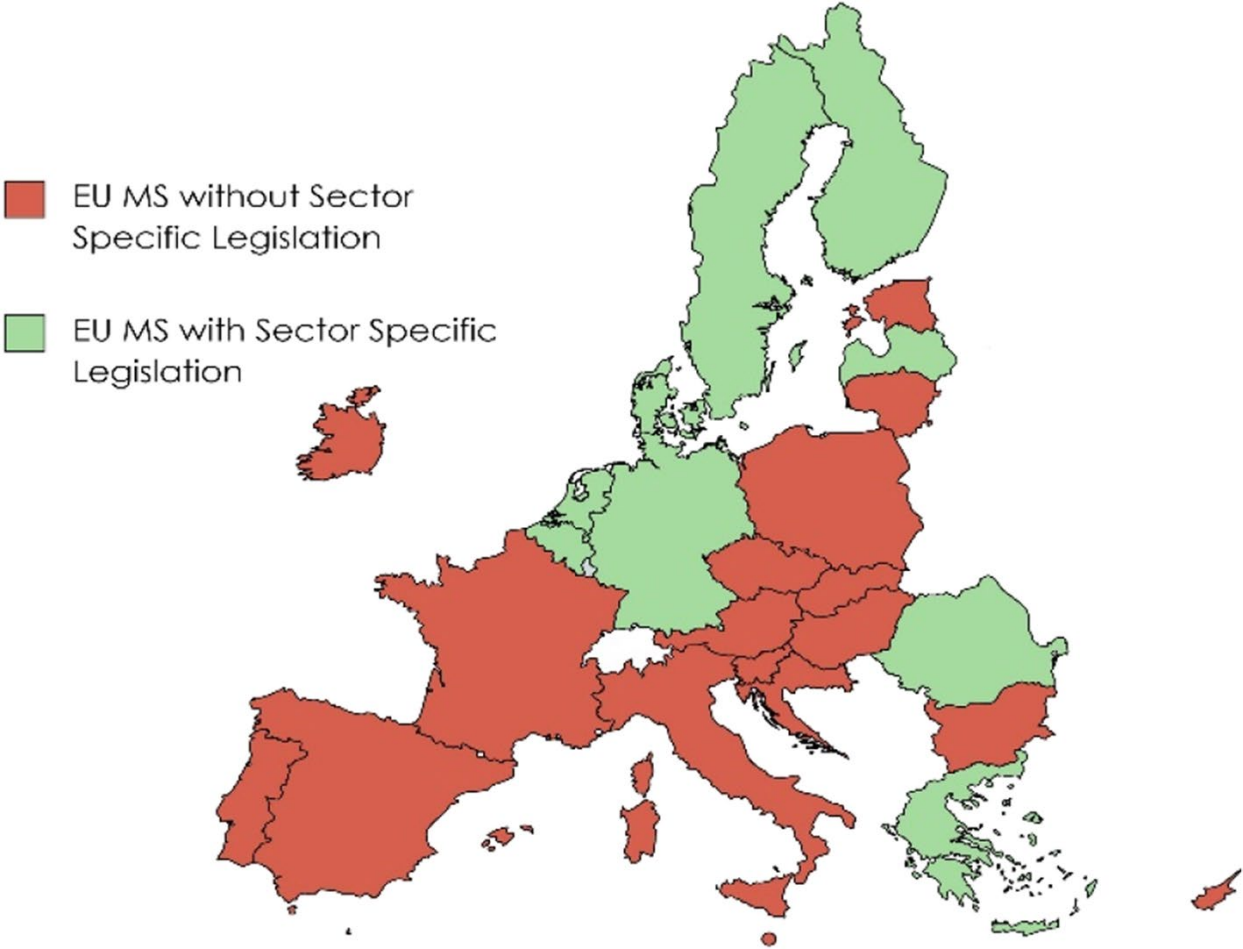
Regulation of abusive debt collection practices in EU MS via sector-specific legislation

Several EU studies and policy papers have found that abusive debt collection must be addressed in a balanced manner. Based on his research of consumer over-indebtedness, Huls established a need to “*protect the debtor and his family from undue demands and harassment by their creditor*, with due regard for the rights of the creditors, whose position can also feel threatened” (emphasis added) (Huls, 1993, p. 220; also cited in Heys et al., 2012, p. 188). Minor references to the fair treatment of consumer-debtors and debt stakeholders’ rights occurred in the European Commission’s (EC) Amnesty of debts: Amicable agreement and Statutory Solution, Minutes (2006). The EC emphasized the “importance of considering the ‘good faith’ of the creditor as well as the debtor, as a way of balancing creditors’ and debtors’ rights” (Heys et al., 2012, p. 188). However, the 2007 Council of Europe’s Final Activity Report of the Group of Specialists for Legal Solutions to Debt Problems soon contradicted this position. The report concluded that “debtors should fulfill their obligations as far as possible” and no “good faith test should be implemented as it is impossible to define good faith” (Heys et al., 2012).

Abusive debt collection made another appearance after the adoption and implementation of the UCPD. In its first Guidance, the EC (2009) included debt collection activities among “after-sale commercial practices regulated by the Directive.” The EC reasoned that “when a consumer owes a trader a certain amount of money (as a consumer debt), the collection of this debt (in-house or by a third party) is directly connected to the sale or supply of products” (EC, 2009). Several MS who used the UCPD to tackle abusive IDCPs endorsed this interpretation. It was restated in the EC’s new Guidance (2016) with the addition that “there are no objective reasons to differentiate that assessment based on whether a trader outsources it (debt collection) through specialized agencies or not.” The CJEU took a similar view in *Gelvora* (2016), a case concerning the legal relationship between a debt collection agency and a consumer debtor, whose debt was assigned to the agency, and whether it falls into the UCPD’s material scope (*infra* Sect The CJEU’s Case Law: an Ancillary or a Quasi-Financial Service?).

Finally, the need to regulate and harmonize abusive IDCPs at the EU level resurfaced in 2020 in connection with the EC’s Action Plan to address NPLs—specifically, the Proposal for a Directive of the European Parliament and of the Council on Credit Servicers, Credit Purchasers and the Recovery of Collateral (CSDP). The CSDP would enable banks to deal more efficiently with non-performing loans, mainly by selling the credit to third parties or outsourcing loan servicing to a specialized debt collector. The proposal harmonizes the rules by which credit servicers must abide to operate across borders within the EU (EC 2018).

Initially, the CSDP contained no harmonized rules against abusive IDCPs other than the licensing of credit servicers even though the EC stated the need for clear rules to protect consumers’ rights and interests (EC 2018). This absence raised the concerns of consumer NGOs and EU advisory bodies ( EESC 2018; Finance Watch 2017; FSUG 2018), and in 2020, several Members of the European Parliament (MEPs) advanced amendments to the CSDP to tackle abusive IDCPs (European Parliament, 2020). The amendments have faced criticism for lacking justification (Huertas & Schelling, 2020). However, the Council of the European Union (2021) announced a compromise text of the CSDP, containing a condensed version of the amendments. If adopted, the CSDP will be the first pan-EU instrument expressly tackling abusive IDCPs.

These developments show that the EU harmonization of abusive IDCP regulation could become a reality, although it is a slow and sinuous process. It raises two issues that legal scholars have overlooked: (1) the multifaceted legal nature of debt collection and its impact on harmonization efforts and (2) the critical assessment of the need to harmonize debt collection regulation at the EU level. The first aspect answers *what* debt collection is and *whether* the EU has the constitutional competency to harmonize debt collection regulation. The second addresses *why* and *how* debt collection should be harmonized.

In this article, I will critically assess the need to harmonize the legal framework concerning abusive IDCPs in the EU, mainly from the standpoint of the CSDP. Where necessary, I will refer to both historical and comparative law perspectives. I focus on the legal character of informal debt collection and its relation to financial services (Sects Comparative Aspects: a Close “Relative”, The CJEU’s Case Law: An Ancillary or a Quasi-Financial Service?, The Credit Services Directive Proposal: Credit Servicing) as well as its potential *sui generis* character (Sect. Debt Collection a Sui Generis Activity; the “what”). After that, I assess the arguments for and against establishing pan-EU sector-specific legislation dedicated to IDCPs (Sects. Arguments for Pan-EU Regulation of IDCPs, Arguments for Maintaining the Status Quo; the “Why”). Next, I discuss the constitutional authority of the EU to regulate abusive IDCPs (Sect. Could the European Union Harmonize the Regulation of Abusive Informal Debt Collection Practices?; the “whether”). Finally, I will examine the interaction of the CSDP with other consumer (financial) protection instruments to identify the best solution for harmonizing abusive IDCPs at the EU level. I will juxtapose several dichotomies: general versus sector-specific (Sect. General or Sector-Specific? (CRD & UCPD v. CSD)), procedural versus substantive (Sect. Procedural or Substantive? (Licenses v. Blacklists)), minimum versus maximum harmonization (Sect. Minimum or Maximum? (MS Autonomy vs. Market Efficiency)), and hard versus soft regulation (Sect. Hard or Soft Law (Can One Trust Self-Regulation)?; the “how”). In the conclusion, I shall synthesize the core problems and suggest an approach.

## Is Debt Collection a Financial Service?

To ascertain whether informal debt collection falls into the ambit of EU regulatory competencies, one must address what informal debt collection is and its relationship to financial services. While the answer to this question might seem apparent *prima facie*, neither EU nor MS legislation or case law are clear. A brief comparison shows that the question is both challenging and significant in other jurisdictions with vast experience regulating IDCPs, such as Australia, the USA, or the UK (until 2016 part of the EU). As the following discussion reveals, whether informal debt collection qualifies as a financial service impacts the availability of protection against abusive IDCPs depending on the origin of the debt.

### *Comparative Aspects: a Close “Relative*”

In Australia, whether informal debt collection is a financial service was raised in *Australian Securities and Investments Commission (ASIC) v Accounts Control Management Services Pty Ltd* (2012) in connection to a debt collection company engaging in abusive IDCPs. The company’s practices violated the 2001 Australian Securities and Investments Commission Act. Since the law required the misleading and deceptive conduct to be “in connection with the supply or possible supply of financial services to a consumer or the payment for financial services by a consumer,” the debt collector argued that its conduct did not meet the threshold because “the business of extracting money from overdue debtors did not involve the provision of a financial service.”

The court rejected the argument because the law qualified loans and credit contracts as financial products, and *the claim of the original creditors was based on such contracts*. In addition, the court held that the debt collector was offering services *in connection* to these products, especially when getting debtors to agree to repayment plans (which resembled ongoing credit). The court pushes the legal boundaries to provide consumers with protection against abusive IDCPs arising from the collection or renegotiation of prior credit agreements. However, it is unlikely that a similar outcome would occur if debts originate from utility bills, which would not involve repayment in installments.

The US Code, Title 12 (Banks and Banking), Chapter 53—Wall Street Reform and Consumer Protection, Section 5481, paragraph 5 defines consumer financial products or services as follows:

Any financial product or service that is described in one or more categories under paragraph (15) and is offered or provided for use by consumers […] or clause (i), (iii), (ix) or (x) of paragraph (15)(A) and is *delivered, offered or provided in connection with a consumer financial product or service*.’ Paragraph (15) (A) (x) is clear that ‘the term financial product or service means […] *collecting debt related to any consumer financial product or service.* (emphasis added)

Despite its apparent clarity, this definition may be misleading. Abusive IDCPs are regulated by a sector-specific, federal act that does not distinguish between the origins of consumer debt (NCLC, 2018, Sect. Hard or Soft Law (Can One Trust Self-Regulation)?*, *Arguments for Pan-EU Regulation of IDCPs). Consumers are protected regardless of whether their obligation arises from financial services or utility bills (NCLC, 2018, Sect. Hard or Soft Law (Can One Trust Self-Regulation)?, Arguments for Pan-EU Regulation of IDCPs). Thus, including debt collection among consumer financial products or services does not limit consumer protection against abusive IDCPs in the USA.

In the UK, the Consumer Credit Act 1974 (CCA 1974) defined debt collection as a type of *ancillary* credit business consisting of taking steps to procure the payment of debts due under credit agreements or consumer hire agreements (CCA 1974, s 145 (7)). Currently, under the Financial Services and Markets Act 2000 (Regulated Activities) Order 2001/SI 2001/544 (RAO) and the subsequent Amendment (No 2, Order 2013), debt collection was brought under the heading “*activities in relation* to debt,” although the definition of debt collection has not changed. Given the exemptions in the amended RAO and CCA 1974, the ambit of debt collection activities covered by the definition remains somewhat limited. However, this does not change debt collection’s legal nature. Moreover, rules related to debt collection are part of credit-related activities (Consumer Credit Sourcebook (CONC) 7.1.1), and so debt collection is treated as a regulated (financial) activity (CONC 7.3). Here as well, the legislator recognizes an intrinsic relationship between credit and debt collection. However, the effects may be limiting. In the absence of additional rules, only debt collection arising from regulated credit agreements is subject to regulation, while collection arising from non-regulated or excluded ones is not.

### The CJEU’s Case Law: An Ancillary or a Quasi-Financial Service?

In the EU, the wording of the existing consumer protection directives does not answer the dilemma. However, the CJEU’s case law on debt collection might hint at one.

In *INKO* (2015), the CJEU considered debt collection agencies that offer installment arrangements to defaulting borrowers on behalf of lenders. The CJEU had to decide whether such agencies fall within the definition of a credit intermediary under Article 3(f) of Directive 2008/48 on consumer credit (CCD). In the specific case, the debt recovery agency was approaching consumer debtors with a pre-printed installment agreement. Under this agreement, consumers acknowledged the debt and agreed to pay it in installments according to a repayment plan and to cover the agency’s remuneration for its services.

In the preliminary remarks of her Opinion (2016), advocate general (AG) Sharpston noted that the CCD does not contain “any information to indicate *whether debt collectors […] supply goods or services acting in an ancillary capacity*” (Para 19) (emphasis added) as set out in Article 7 of the directive. Moreover, AG Sharpston observed that how the legal arrangement between lenders, borrowers in default, and debt collection agencies were to be classified, and the applicable rules differed across MS (Para 20), assuming that such rules were in place and were clear enough to provide an answer.

In the AG’s view, a debt collection agency providing such services would qualify as a credit intermediary under CCD, a position similar to that of the Federal Court of Australia. To justify her interpretation, the AG juxtaposed the legal provisions with the case’s facts. She found that if the debt collection agency concludes credit agreements with consumers on behalf of the creditor, the wording is sufficiently broad to cover the debt collector’s activities (Para 26). In addition, she argued that such an interpretation would follow the twin aims of consumer protection and create a genuine internal market, as envisioned by the CCD (Para 27). In the AG’s opinion, the practice of providing defaulting consumers with 3 days to assess their position was inadequate. The choice between full payment and entering the installment agreement was unlikely to be genuine (Para 28). In this article’s view, what AG Sharpstone noticed was an abusive IDCP, given the undue pressure it would put on the consumer. The AG concluded that while “debt collection is not an activity associated with a more conventional type of credit intermediary,” the term is a general one and covers several types of activities (Para 33).

In its decision, the CJEU sided with the AG. First, it noted that regarding consumer credit, the CCD sought to establish full and mandatory harmonization in several key areas to ensure a high and equivalent level of protection for all EU consumers (Para 27). It also endorsed the AG’s opinion that the concept of the credit agreement “is particularly broad and covers an agreement […] which provides for the rescheduling of repayments of an existing debt” (Para 30), which helps to meet the objective of consumer protection (Para 35). The CJEU concluded that a collection agency that acts on behalf of the lender to collect an unpaid credit, interest, and costs must is as a “credit intermediary” under the CCD (Para 44).

Nevertheless, it remains unclear whether debt collection qualifies as a financial service. Although debt rescheduling is part of a debt collector’s arsenal, it is still an ancillary instrument to an original (financial) product, which brings it into the ambit of the CCD. However, most other debt collection practices would not qualify as services or products under the CCD since not all consumer debt arises from credit agreements.

In *Gelvora* (2016), the CJEU considered two questions. First, whether the legal relationship between a private debt collection agency and a debtor whose defaulted debt was assigned to that agency falls within the UCPD’s scope. Second, whether the term product, as defined by UCPD, Art 2 c), encompasses the company’s recovery practices (Para 18). One must note here that the concepts of commercial practices/products are broader in scope than that of credit agreements. Even so, the court found a link between the two.

In its analysis of the concept of “product,” the CJEU notes that “the claims assigned to Gelvora *originate in the supply of* a service, namely the provision of *credit*” (emphasis added) (Para 22). This reasoning brings it close to that of the Australian federal court mentioned above. However, the court avoids providing a clear answer to whether debt collection is a financial service. It states that “although a debt collection agency […] does not provide the consumer with a consumer credit service *as such*, the fact remains that the activity […] falls under the concept of commercial practices” (Para 25) (emphasis added).

The conclusion is that debt collection may still qualify as a *quasi*-financial service even if it cannot be considered a financial service *proper*. By equating it to an “after-sale commercial practice” and considering all the elements of the UCPD’s definition, debt collection is “*directly connected with* the promotion, *sale or supply of a financial product* to consumers” (UCPD, Art 2 d) (emphasis added). The court explains that if the UCPD did not apply to abusive IDCPs, this would discourage consumers from entering credit agreements (Para 27), underlining the inextricable link between financial services and IDCPs again.

Nevertheless, these cases are linked to the provision of consumer credit, and their application to consumer debt not originating in financial services remains doubtful.

### The Credit Services Directive Proposal: Credit Servicing

In 2018, the EC advanced the CSDP, a document intended to standardize the regulatory regime (i.e., the definition, authorization, supervision, and conduct rules) for credit servicers and purchasers. If adopted, the 2021 version of the CSDP would introduce credit servicers and credit service providers as a new category of EU service providers*.* Credit servicers are “legal persons who, in the course of its business, manage and enforce the rights and obligations related to the creditor’s rights under a non-performing credit agreement, or to the non-performing credit agreement itself, on behalf of the credit purchaser and carry out at least one or more credit servicing activities” (Council of the EU, 2021, Art 3 (7b)). Credit service providers are “third parties used by a credit servicer to perform any of the credit servicing activities” (Council of the EU, 2021, Art 3 (7a)). It is beyond the scope of this article to discuss the questionable division between credit servicers and credit service providers (which will be addressed in a future commentary). Suffices to say that the current wording of the CSDP seeks to cover professional debt collectors, acting either as principals or as agents.

Of greater relevance is the definition of credit servicing activities. It encompasses “collecting or recovering from the borrower […] any payments due related to a creditor’s rights under a credit agreement or to the credit agreement itself” and “renegotiating with the borrower […] any terms and conditions related to a creditor’s rights under a credit agreement or of the credit agreement itself in line with the instructions given by the credit purchaser,” where the credit servicers is not a credit intermediary, as defined by the CCD (Council of the EU, 2021, Art 3 (7c) (a) and (b)).

While the CSDP does not solve the dilemma of whether debt collection is a financial service, it acknowledges debt collection’s intricate connection with financial services such as consumer credit. Thus, the CSDP proposes another way of looking at the problem: Debt collection is neither a proper nor a quasi-financial service. Instead, it is a *specific* service (e.g., the recovery and renegotiation of debts) linked to a particular financial product (e.g., consumer credit agreements). This view is limiting because it connects debt collection to consumer credit agreements, which is not always the case. Consumer debt can also stem from sources other than credit transactions such as utilities, rent, insurance bills and claims, attorney fees, or judgments. Moreover, both the CCD and the Directive 2014/17 on consumer agreements relating to residential immovable property (CMD) exclude a wide variety of transactions from their scope, which would enable them to escape compliance with the CSDP’s requirements.

### Debt Collection a Sui Generis Activity

**Fig. 2:**
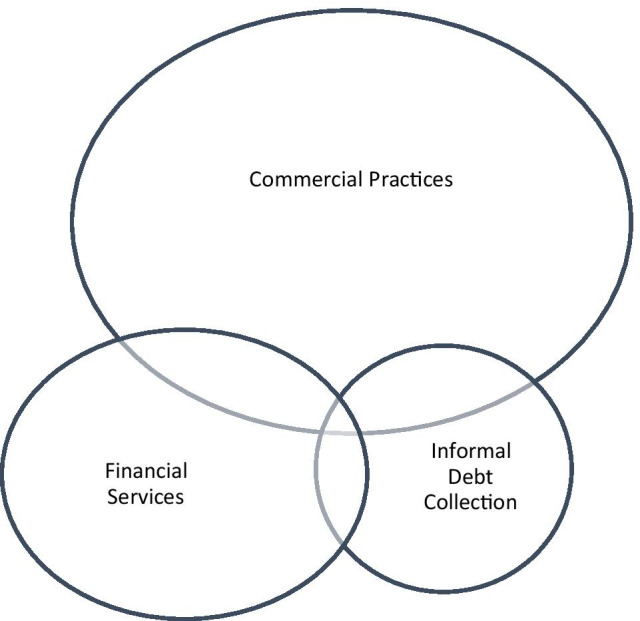
Overlaps between commercial practices, financial services, and informal debt collection

The national approaches of EU MS also fail to provide a clear understanding of informal debt collection’s legal nature. The definitions extracted from sector-specific legislation addressing IDCPs suggest that informal debt collection could be a *sui generis* category of *independent professional* services (Table 1) that is (a) only tangentially connected with financial services and credit servicing and (b) only partially overlaps with commercial practices (Fig. 2).

**Table 1:** Definitions of Informal debt collection in the Nine EU MS with sector-specific regulation of abusive IDCPs

Country	Definition of informal debt collection
Belgium	“Any act or practice whose purpose is to determine the debtor to pay an unpaid debt, except for any recovery based on an enforcement title,” while the “activity of amicable debt recovery is the **professional** activity, undertaken either mainly or ancillary, by a natural or legal person consisting in the amicable recovery of debts unpaid by a third party, without having contributed to the conclusion of the original contract, as well as the amicable recovery of debts assigned in exchange for remuneration”
Denmark	**Professional** recovery of debts for others or of debts that have been purchased, after the due date, for the purpose of recovery for oneself
Finland	The recovery of receivables on behalf of another, as well as the recovery of own receivables, in cases where it is obvious that receivables have been received exclusively for recovery
Germany	Collection of debts or assigned debts for recovery on behalf of a third party, performed as an **independent operation** (collection service) is an extra-judicial legal service
Greece	The totality of extra-judicial approaches performed by companies for the information of debtors concerning their due debts toward creditors, which are arising from contracts and other legal documents, such as loan agreements, invoices, bills, shipping notices, that govern the relationship between creditor and debtor
Latvia	An aggregate of extra-judicial activities used by a creditor or provider of debt recovery services, inviting a debtor to carry out the delayed payment obligations voluntarily
The Netherlands	Extra-judicial collection activities for the repayment of an amount of money
Romania	N/A
Sweden	Measures taken to put pressure on the debtor other than granting additional time for payment or the notice that, in case of non-payment, the debt will be assigned to a third party for recovery

To further clarify the matter, it is also necessary to summarize the types of debt that can be recovered via IDCPs, as not all national laws have the same scope and coverage.

In Denmark, the recovery of own receivables—own collection—does not fall into the law’s ambit (with some exceptions). Otherwise, it appears that *all types of debt*—commercial, consumer, or obligation arising from agreements among natural persons who do not act in their professional capacity—are covered, notwithstanding their origin (e.g., credit agreements or utilities). A similar approach is identifiable in Sweden, under the condition that debt originating from agreements between natural persons is related to economic activities or assigned for collection. Greece specifically lists among creditors: credit institutions, insurance companies, utility companies, telecommunication companies, retailers, and service providers. Although Germany does not specify which type of debt can become the object of extra-judicial recovery, it is among the countries that allow collecting all kinds of debt.

In Finland, Belgium, Latvia, the Netherlands, and Romania, the laws cover *only consumer debt* from a hire-purchase agreement or a credit agreement. However, in Finland, consumer credit is broadened to include loans between natural persons.

These differences reveal that debt collection has a rather distinct nature (*sui generis*) and varies in coverage depending on both the active subject (creditors and debt collectors) and the passive one (the debtor). The laws of Denmark, Sweden, Greece, and Germany stand in stark contrast with the EU’s approach, which limits the protection to situations arising from consumer credit agreements. Thus, any attempt to categorize debt collection either as a financial service or credit servicing would also be limiting. Given the existing cross-border dimension of IDCPs (Stănescu, 2021) and its envisioned expansion, once the CSDP is adopted, such limitations may negatively impact the harmonizing intervention of the EU in regulating abusive IDCPs.

## Should the European Union Regulate Abusive Informal Debt Collection Practices?

The first question that arises is whether the EU should regulate IDCPs. To answer it, the article presents a list of arguments for and against EU intervention. As the subsequent analysis will reveal, the former outweigh the latter.

### Arguments for Pan-EU Regulation of IDCPs

Many factors justify the EU’s intervention in regulating abusive IDCPs. *First,* abusive IDCPs are now a common issue in the EU, with a significant cross-border dimension. Two EU-wide surveys from 2020 revealed that abusive IDCPs are rampant across the union (Jérusalmy et al., 2020; Stănescu, 2021). In one of the surveys, 17 of 20 respondent EU MS specified that they had received complaints regarding abusive IDCPs in the past five years. Moreover, ten of the 17 EU MS respondents indicated that the complaints concerned foreign debt collectors, and the actual numbers of MS with such complaints could be higher (Stănescu, 2021).^1^

*Second,* two-thirds of the EU MS do not regulate abusive IDCPs at the national level via sector-specific legislation (Fig. 1). According to Stănescu’s survey (2021), only nine EU MS, totaling around one-third of the EU’s population, have implemented such legislation. The reality undermines both the idea that abusive IDCPs are a local matter and the concept of the level playing field for debt collectors and consumers in the EU, opening the way for regulatory arbitrage.

*Third*, the existing national legal frameworks—both sector-specific and general traditional remedies—display (with some exceptions) heterogeneous and idiosyncratic features that affect their viability (Council of the EU, 2021, Recital 13; Stănescu, 2015), especially in tackling cross-border abusive IDCPs and enforcement (Stănescu, 2021).

*Fourth,* there are no common minimum standards at the EU level concerning the regulation of abusive IDCPs, as acknowledged by the European Parliament (2020) and Recital 13 of the current version of CSDP (Council of the EU, 2021). The Directive 2011/83/EU on consumer rights (CPD) or the Directive 93/13/EEC on unfair terms in consumer contracts (UCTD) do not affect abusive IDCPs. Moreover, the UCPD plays a marginal, ancillary role (Stănescu, 2020, 2021), even if some MS have used it to tackle abusive IDCPs (EC 2009, 2016; Stănescu, 2020, 2021) and the Gelvora (2016) has endorsed its application to post-sale commercial practices, such as IDCPs. The UCPD did not intend to cover abusive IDCPs comprehensively. Out of 23 blacklisted items, only two have application in debt collection: Claiming a service or a product was approved or authorized by a public body and falsely claiming or creating the impression that the trader is not acting for the purposes relating to her trade, business, craft, or profession (Stănescu, 2020). It contains no rules concerning the authorization of debt collectors and no rules concerning the validation of the debt or the issue of added charges to the original debt, and, until the adoption of the Enforcement Directive 2019, it provided no individual remedy or private right to action to aggrieved consumer-debtors (Stănescu, 2020).

Moreover, additional issues such as the conditions regarding the consequences of the unfair IDCPs and the compliance with the average consumer standard impair its application to debt collection (Stănescu, 2020). Finally, although it is a maximum harmonization instrument, the UCPD allowed EU MS to implement additional rules for consumer financial protection, including IDCPs. The UCPD thus acknowledged both the importance of financial security for consumers and its insufficiency in addressing it (Recital 10 and Art 3.9) (Stănescu, 2020).

*Fifth,* in their amendments to the CSDP, several MEPs called on the EU MS to “ensure that behavior or practices that are likely to impact on borrower privacy and/or human dignity or are likely to mislead them are prohibited” (European Parliament, 2020). The MEPs observed that abusive IDCPs are likely to “aggravate the borrower’s situation” and called for the introduction of banned practices and common standards of behavior in informal debt collection in the text of the CSDP (European Parliament, 2020). The 2021 version of the CSDP states that credit servicers (e.g., debt collectors) “should always act in good faith, treat borrowers fairly and respect their privacy” and “should not harass nor give misleading information to borrowers” (Council of the EU, 2021, Recital 20a).

*Sixth,* policy considerations support regulating abusive IDCPs at the EU level. The Treaty on the Functioning of the EU (TFEU) requires consumer protection to be considered when defining other EU policies. It also states that EU consumer policy seeks to promote (among others) consumers’ health, safety, economic interest, and their right to information […] and to organize themselves to protect their interests. In the aftermath of the 2008 financial crisis, the EC reemphasized the need to protect consumers in the European Consumer Agenda – Boosting Confidence and Growth (2012). Among other issues, the EC noted that many problems faced by consumers are not remedied because consumers do not act, mainly when the sums involved are small. The EC also observed that cross-border situations hamper enforcement of consumer rights. While the EC also stressed the importance of the financial services sector, it did not even consider abusive IDCPs.

To date, the EU has adopted no sector-specific legislation to protect consumer-debtors against abusive IDCPs. On the contrary, until recently, the UCPD—which, in the view of the EC and the CJEU, covers unfair debt collection practices and aims to “directly protect consumer economic interests”—provided no right of action to individual consumers if they became victims of abusive IDCPs. The UCPD also states its intention to “indirectly protect legitimate businesses from their competitors who do not play by the rules in this Directive and thus, guarantees fair competition in the fields coordinated by it” (Recital 8), thus removing the distortion of competition (Stuyck et al., 2006). Along the same lines, the European Consumer Agenda (European Commission, 2012) highlighted the need to ensure that “unfair trading practices do not bring a competitive advantage.” The Agenda stressed that since consumers face services across borders more frequently, improving the regulatory framework on service safety and enhancing market surveillance is necessary. To address all these concerns, the EU should implement legislation dedicated to informal debt collection. It remains to be seen if and to what extent the 2021 version of the CSDP will achieve this goal.

*Finally,* empirical data collected via a 2020 EU-wide survey reveals that all respondents—both regulatory agencies and debt collection associations—agreed that abusive IDCPs are unacceptable and should be tackled, preferably with more precise regulation (Jérusalmy et al., 2020; Stănescu, 2021). In the opinion of the Belgian regulator, “it would be great if this legislation might be applicable all over Europe.” At the same time, the German debt collectors’ association stressed that the industry’s Code of Conduct should be turned into law, thus making it mandatory for all debt collectors (Stănescu, 2021). The fact that the 2021 version of the CSDP specifically tackles abusive IDCPs indicates that the EU legislators felt such rules are needed.

### Arguments for Maintaining the Status Quo

One can raise several objections against the regulation of abusive IDCPs by the EU.

*First,* one may argue that abusive debt collection practices are an internal issue to be handled by the EU MS in line with the fundamental freedoms and that EU intervention would affect MS autonomy. AG Maduro partly used this line of thought in *Commission v Italy* (2005). His Opinion (2006) stated that while “it is no doubt desirable that [extra-judicial debt recovery] should be regulated” (Para 9), the states should not use conditions that would be too restrictive to the pursuit of activity across the EU.

Empirical data refutes this argument because regulation at the MS level is more of an exemption than a rule (Stănescu, 2021), as shown in Sect. Arguments for Pan-EU Regulation of IDCPs. The EU legislators acknowledge this fact in the 2021 version of the CSDP (Council of the EU, 2021, Recital 10). In addition, debt collection activities are no longer an internal matter, and the cross-border nature of collection will only increase once the CSDP comes into force. The CSDP attempts to level the playing field for credit servicers (including debt collectors) by harmonizing licensing and authorization requirements across the EU. However, fostering the development of the single financial market and completing the Banking Union does not fully curb the regulatory autonomy of the MS (Micklitz in Grundmann and Micklitz, 2019). Suppose the 2021 version of the CSDP is adopted, EU MS will retain the possibility to regulate credit servicing activities that do not fall within the scope of the Directive (Council of the EU, 2021, Recital 16a).

*Second,* one may argue that the existing regulatory framework—the UCPD—and the original CSDP suffice in tackling abusive IDCPs. The UCPD applies to debt collection based on an extensive interpretation of its scope, which refers to “unfair business-to-consumer commercial practices, […] after a commercial transaction in relation to a product.” This interpretation has already enabled several EU MS lacking sector-specific legislation to punish some misleading and aggressive debt collection practices (EC, 2016), and the CJEU endorsed it in *Gelvora* (2016). The court emphasized that an interpretation to the contrary would encourage service providers to separate the recovery of payment from the service of providing credit, which would undermine consumer protection. However, as detailed in Sect.  Arguments for Pan-EU Regulation of IDCPs, the UCPD’s application to abusive debt collection practices is limited (Stănescu, 2020).

The initial CSDP indirectly acknowledged the need to protect consumers against abusive IDCPs, by encompassing requirements about the integrity and professionalism of credit servicers (including debt collectors) (EC 2018, Recitals 24 and 53 corroborated with Art 5). However, these provisions could hardly replace adequate sector-specific legislation or common standards of good conduct applicable to the internal market.

The Regulatory Scrutiny Board of the EC (2018) criticized the Impact Assessment Report to the CSDP because the report did not “sufficiently examine how the initiative might affect debtors, data protection and other national legal provisions.” The suggested amendments to the CSDP emphasized the need for good faith and fair and respectful treatment of consumers and their privacy by blacklisting abusive practices; they also affirm the role of the MS in ensuring compliance with a set of minimum EU common standards for debt collection (European Parliament, 2020, Amendments 314, 336).

The 2021 version of the CSDP alleviates most of these concerns. It maintains the initial requirements about the integrity and professionalism of credit servicers (including debt collectors) (Council of the EU, 2021, Recital 24a corroborated with Art 5) and adds a minimum set of rules of conduct (Council of the EU, 2021, Recitals 20a, 21 and 21a corroborated with Art 8a). These measures help exclude some unscrupulous agents from the industry and implement *de minimis* common standards of behavior throughout the internal market.

*Finally,* one may object that EU regulatory intervention against abusive IDCPs lacks supporting empirical data. In March 2020, an article on the status of CSDP’s implementation noted that the proposed amendments regarding abusive IDCPs “contain very few explanatory statements on the reasons for the said amendments” (Huertas & Schelling, 2020). However, recent empirical studies provide support to the proposed amendments. Following their survey conducted in 23 European countries, Jérusalmy et al. (2020) called for a minimum level of common EU standards for debt collection when dealing with consumers and a common EU list of prohibited activities. Stănescu (2021) made a similar recommendation following an EU-wide survey covering 26 EU MS.

This section has presented arguments for and against the EU’s intervention in regulating IDCPs. It found sufficient empirical support for the EU legislator to promote and implement adequate regulation of abusive IDCPs across the union and evidence that such legislation might become a reality if the CSDP is adopted. In other words, the arguments for regulation outweigh those for the *status quo.* However, a common IDCP legal framework raises questions about harmonization and whether the EU has the constitutional competence to intervene. The following section seeks to address these questions.

## Could the European Union Harmonize the Regulation of Abusive Informal Debt Collection Practices?

A discussion of whether the EU can regulate (and harmonize) abusive debt collection practices must address the ongoing establishment of the internal market (Craig and de Búrca 2020), the issues related to EU’s competence to harmonize, and (most importantly) the regulatory competition between the EU and the MS (Chalmers et al., 2010).

The internal market aims to merge MS markets into a single larger market, which requires more uniformity of structure and conditions. The CSDP states its purpose of completing the Banking Union and establishing all the building blocks for a Capital Markets Union (Council of the EU, 2021, Recital 2), including well-developed NPL markets (Macchiarelli et al., 2019). The CSDP would foster the development of secondary markets for NPLs by first removing impediments to the transfer of NPLs to non-credit institutions, followed by simplifying and harmonizing authorization requirements for credit servicers. The CSDP would establish an EU-wide framework for both the purchasers and servicers of credit agreements (Council of the EU, 2021, Recital 24). This appears to be in line with the EC’s New Approach to harmonization (EC 1985, para 102), which states that “using a minimal coordination of rules (especially on such matters as authorization, financial supervision and reorganization, winding up)” should suffice as a basis for mutual recognition among MS.

Given the potentially harmful effects for EU consumers, the EC’s desire to facilitate and foster cross-border transactions of NPLs unavoidably entails involvement in matters of broader social concern. It should not constitute an obstacle to the EU’s intervention. There are precedents such as the working conditions of workers or the quality of foodstuffs (Craig & de Búrca 2020). Policy considerations in the EU MS and around the world reveal that unrestricted abusive IDCPs have detrimental effects on consumers’ physical, psychological and economic well-being (Warren et al. 2014; Stănescu, 2015), fair competition (UCPD, Recital 8), and the financial services industry (Gelvora 2016).

The general coverage for creating the internal market starts with Art 26 of TFEU, which empowers the EU to adopt rules on matters affecting interstate trade, such as standards or consumer rights. Art 26 also describes the internal market as an area without frontiers and without obstacles to the free movement of goods, persons, services, or capital, which are the two conditions needed to complete the internal market (Craig & de Búrca, 2020; Micklitz in Grundmann and Micklitz, 2019). Credit servicing—including both debt purchase and debt collection—is a perfect test subject because its regulation would affect all four freedoms and has a considerable social policy dimension.

An essential question is what would constitute a hindrance to cross-border (intra-EU) informal debt collection. The widely accepted interpretation is that only measures that make movement across borders more difficult than staying at home or discriminate against foreign persons are covered. In one view, the restriction on movement between MS necessitates some comparison to establish the existence of a specifically trade-negative effect (Chalmers et al., 2010). In another, any comparison would be irrelevant, and restrictions on cross-border movement should be covered despite equivalent domestic effects unless they are reasonable and would amount to a general proportionality review of national law affecting economic activity (Chalmers et al., 2010). It is not the purpose of this article to endorse or invalidate the two positions; both can find justification for the regulation of intra-EU abusive IDCPs.

### The Comparison Theory

In *Commission v Italy* (2005), the CJEU used the comparison test. Although a hindrance was present, it was deemed proportional and justified by the general purpose of protecting public interests and ensuring close supervision of extra-judicial debt recovery activities (Paras 30–31).

The dispute arose when Italy made extra-judicial debt recovery subject to occupational licensing. As AG Maduro pointed out in his Opinion (2006), states enjoy the freedom to regulate professions, and they introduce obstacles to freedom to provide services in the absence of harmonization at the EU level. However, such measures should still comply with the principle of proportionality (Paras 6, 13, and 15). The EC and the AG issued a joint opinion that by implementing licensing requirements for debt collectors from other MS, Italy had breached EU law by restricting the freedom to provide services.

The EC raised eight issues about the conditions and obligations imposed by the legislation in force in Italy for pursuing extra-judicial debt collection activities. The first was the incompatibility of the requirement of obtaining a license with Art 49 EC. An affirmative answer from the CJEU would have meant an almost “free-for-all” type of debt collection business on the internal market.

However, in its judgment (2007), the court took a different stance and rejected the EC’s claim and the AG’s position. It started by admitting that in its previous case law, it clarified that the national legislation that makes the provision of services within national territory by an undertaking established in another MS subject to an administrative authorization constitutes a restriction on the freedom to provide services under Art 49 EC (Paras 23–25). Seen solely from this perspective, legislation such as the one in question would have been, in principle, contrary to EU law, especially if MS did not consider the obligations with which the provider of cross-border services must comply in its home state.

However, the court also acknowledged that MS could impose restrictive requirements on the grounds of public interest. The court held that in the Italian legislation, the practice was simply to require a declaration of “good conduct” to be submitted via the Internet to the authority. This requirement was considered less demanding than the obligation to supply documents to the competent authority. Therefore, in the view of the court, one could not argue that the procedure failed to consider the service provider’s compliance with the legislation in the state of origin (Paras 28–29).

Consequently, the CJEU ruled that Italy did not go beyond what was necessary to attain the objective of ensuring the close supervision of extra-judicial activities. Moreover, it found that the practice was in accordance with the principle of proportionality, as it was justified by reasons related to the public interest (Paras 30–31).

The following question would be whether the EU can harmonize the authorization of undertakings within a specific industry (such as debt collection) by subjecting them to the same requirements, notwithstanding the MS of registration. Based on the financial-services-related contents of the 1985 White Paper on Completing the Internal Market (EC 1985) and the provisions of the 2021 text of the CSDP (Council of the EU, 2021, Recitals 13, 16, 16a, and 24), the EC’s position appears to be affirmative.

### The Hindrance Theory

The hindrance theory is also easily recognizable in connection to the legal framework under scrutiny. The CSDP states that credit purchasers and credit servicers cannot reap the benefits of the internal market “due to barriers erected by divergent national legislation in the absence of a dedicated and coherent regulatory and supervisory regime” (Council of the EU, 2021, Recitals 10 and 13). Rather than a particular rule, the obstacle consists of the differences existing between national legal frameworks. These differences make pan-EU compliance difficult and discourage credit purchasers from operating in more MS, leading to low demand, weak competition, and low bid prices for NPL portfolios (Council of the EU, 2021, Recitals 11 and 15). Thus, the CSDP proponents argue that “there is a clear Union dimension to the development of markets for credits” (Council of the EU, 2021, Recital 11; Explanatory Memorandum to CSDP, p. 2).^2^

At least at a declarative level, consumer protection is not expected to suffer from creating a pan-EU legal framework for credit servicers. The CSDP states it repeatedly: “the assignment of the creditor’s rights […] to a credit purchaser should not affect the level of protection granted by union law to consumers in any way” (Council of the EU, 2021, Recital 18); “it is important that Union and national consumer protection rules continue to apply” (Council of the EU, 2021, Recital 33); “credit purchasers that enforce the purchased credit agreement directly should do so in compliance with […] consumer protection rules applicable to the borrower” (Council of the EU, 2021, Recital 36).

Until the compromise text of 2021, the problem was that “consumer protection” referred solely to the general or national rules already in place, mainly the defenses arising from or connected to the initial credit agreement (Recital 33). The current version of the CSDP now considers that a harmonized, pan-EU market for debt collectors requires a harmonized, pan-EU ban of abusive IDCPs.

The initial text of the CSDP contained no such rules, although it acknowledged both the risks (EC, 2018, Recital 24) and that only one-third of EU MS provide sectorial protection (EC, 2018, Recital 36). The 2018 CSDP would only harmonize the conditions for granting and maintaining authorization for credit servicers. It requested that they have “a clean police record concerning serious criminal offenses linked to crimes against property, to crimes related to financial activities and crimes against the physical integrity and that they are of good repute” (EC, 2018, Recital 24). These were both an explicit acknowledgment that debt collection may take a turn for the worse and a precaution against collectors disposed toward abusive practices. Initially, the CSDP only required debt collectors to “act fairly,” a broad but vague term that encompassed an obligation to abstain from unfair practices (such as those arising from the UCPD) (EC, 2018, Recital 24). The 2021 version, however, takes a broader approach. It requires debt collectors to act in good faith, to treat debtors fairly and to respect their privacy, and to refrain from harassing, coercive or misleading behavior, even if the details are left to the MS (Council of the EU, 2021, Recitals 20a and 21 corroborated with Art 8a).

In summary, the EC took a minimalistic approach in the CSDP. To foster the creation of NPL secondary markets, the EC harmonizes the requirements for registration and occupational licenses by creating a unique framework for carrying out credit servicing activities (Council of the EU, 2021, Recital 9) and implementing a *de minimis* set of banned abusive IDCPs. Thus, it is safe to conclude that there are no constitutional obstacles to harmonization, assuming sufficient political will.

## How Should Abusive Informal Debt collection Practices Be Regulated in the European Union?

Once established that harmonization is plausible, the question becomes how best to achieve it. Several options are available depending on whether the EU legislator (a) chooses a general or a sector-specific instrument; (b) prefers a hard- or soft-law approach; (c) focuses on procedural or substantive rules, and (d) aims for minimum or maximum harmonization.

### General or Sector-Specific? (CRD & UCPD v. CSD)

The EU consumer protection framework is diverse and consists of many legislative documents, which vary in their degree of specificity. Thus, one may wonder which type of instrument would be the most appropriate for harmonizing the regulation of abusive IDCPs in the EU.

Among the documents with *general coverage* are the framework directives: the CRD, the UCTD, and the UCPD.^3^ Despite being the general framework for consumer protection, the CRD does not apply to financial services (CRD, 2011, Recital 32 and Art 2. (d)), which are defined broadly as “any service of a banking, credit, insurance, personal pension, investment or payment nature.” The UCPD excludes financial services from its maximum harmonization goal due to their complexity and inherent high risks to consumers (UCPD, 2005, Recitals 9, 10 and Art 3.9). The exclusion explains why nine MS could implement or maintain sector-specific legislation on abusive IDCPs, although the EC claimed the UCPD covers them. The documents with *specific coverage* include the CCD, the CMD, and the CSDP. Therefore, there does not seem to be an obstacle in implementing sector-specific legislation regarding abusive IDCPs via either the CSDP or another dedicated instrument.

The discussion above suggests that the EU legislator prefers specific instruments for regulating financial (or related) services. If this is the case, abusive IDCPs should also be regulated via a sector-specific instrument. Since the current wording of the CSDP considers debt collection to be a credit service activity, the regulation of abusive IDCPs belongs in the CSDP. If the 2021 version of the CSDP were adopted, the level playing field and protection of EU consumers against abusive IDCPs would become a reality across the union.

However, debt collection does not occur only in connection to consumer credit. Thus, the application of the CSDP to all abusive IDCPs would be limited, an aspect acknowledged by legislators who leave it up to the MS to regulate activities outside the directive’s scope (Council of the EU, 2021, Recital 16a). The greatest danger stems from the absence of overall coherence in consumer protection, which has become more and more fragmented. Should the CSDP be chosen as a sector-specific instrument for regulating credit servicing, including abusive IDCPs, it would still leave out many abusive IDCPs unrelated to consumer credit. There would still be an overlap between banning certain misleading IDCPs (via CSDP) and unfair commercial practices (under UCPD), which must be decided by the *lex specialis derogate legi generali* principle (EC, 2013).

To address these shortcomings, I postulate that the EU should regulate abusive IDCPs via a dedicated instrument to cover all consumer-related debt collection without limiting itself to activities related to or arising from consumer credit. While this would require significant political will and effort, it would provide cohesion and certainty across the EU for all stakeholders, including creditors, supervisory agencies, debt collectors, and consumers.

### Procedural or Substantive? (Licenses v. Blacklists)

Could the EU harmonize licensing or substantive requirements for debt collectors across the union? As shown in Sect. Could the European Union Harmonize the Regulation of Abusive Informal Debt Collection Practices?, the answer seems to depend solely on political will. In the CSDP, there are rules for “*simplifying and harmonizing the authorization* requirements for credit servicers” (emphasis added). These would establish an EU-wide framework for both purchasers and servicers of credit agreements issued by credit institutions.

According to the CSDP, the need to harmonize authorization requirements stems from the existing “barriers erected by divergent national legislation in the absence of a dedicated and coherent regulatory and supervisory regime” (Council of the EU, 2021, Recital 10). The CSDP notes that “Member States have very different rules for how non-credit institutions may acquire credit agreements from credit institutions,” some being unregulated, while others are subject to various requirements. According to the proponent, these differences have resulted in “considerable obstacles” in cross-border debt purchases within the EU, and the solution is to “harmonize the authorization procedure” (Council of the EU, 2021, Recital 10).

The EU legislator observes that the uniform and harmonized authorization of credit servicers would help promote trust and avoid a reduction in debtor or borrower protection. Authorization procedures would ensure a level playing field for competition among credit servicers and serve as the first line of defense against abusive IDCPs. State supervision would indirectly protect consumers by screening and removing criminal elements. Thus, the CSDP calls for authorization rules to ensure that service providers.

have a clean police record in relation to serious criminal offenses linked to crimes against property, to crimes related to financial activities, or to crimes against the physical integrity and that they are of good repute. Similarly, these persons, as well as the credit servicer, should not be subject to an insolvency procedure or have not previously been declared bankrupt unless they have been reinstated in accordance with national law. Finally, to ensure compliance with debtor protection as well as personal data protection rules, it is necessary to require that appropriate governance arrangements and internal control mechanisms and recording and handling of complaints are established and subject to supervision. Moreover, credit servicers should be obliged to act fairly and with due consideration for the financial situation of the borrowers.

Under the 2021 version of the CSDP, debt collectors qualify as credit servicers or credit service providers (Council of the EU, 2021, Art 3(7a) and 3(7b)). Thus, the conditions listed by the CSDP to authorize credit servicers would also apply for licensing debt collectors.

In light of the CSDP, persons with criminal records involving violence or fraud should be precluded from credit-related services, including debt collection. This requirement is present in most MS that license debt collectors.^4^ Measures should be in place to insulate consumer debtors from exposure to criminal behavior and ensure that their privacy and personal data are protected and that they will not be subjected to any unfair or abusive practices. While no such harmonized authorization procedure for debt collectors currently exists at the EU level, the CSPD provides a plausible pathway for adopting and implementing such a measure.

Despite the above, a harmonized licensing or authorization procedure alone would not adequately protect consumer-debtors against abusive IDCPs. The CSDP states that “credit servicers *should always act in good faith, treat borrowers fairly and respect their privacy*” (emphasis added), which implies that it will be a task for the MS to design and implement national rules to enforce these obligations. MS will need to regulate abusive IDCPs at the national level. While such a solution might please the MS and allow them to retain their regulatory autonomy, it will impact the very purpose of the CSDP. Credit servicers would need to comply with a standardized authorization procedure but may face a very fragmented legal framework, making them reluctant to operate across borders. Such an approach will create uncertainties for consumers, for they will enjoy different protection standards from one MS to the other.

The Regulatory Scrutiny Board (2018) noticed the danger hidden in the CSDP, stating in a negative opinion that the draft impact assessment report:

should clarify the trade-offs between the positive impacts for creditors through the development of secondary markets on the one hand, and potentially negative impacts this might have in terms of debtor protection, data protection, consumer protection or fundamental rights. The report should better detail the conditions/rules to apply to investors and loans servicers (in particular vs. banks) and the potential or additional safeguards for debtors.

The 2018 version of CSDP contained no such rules. It is why several MEPs advanced amendments to regulate the activities related to debt collection, noting the absence of “common standards” at the European level (European Parliament, 2020, Amendments 229–230) and advocating for the introduction of “minimum EU common standards for debt collection” (European Parliament, 2020, Amendments 336–338 and 350). The proposed minimum standards were substantive and included rules about debt validation, mandatory prior notification (based on a standard EU template), and the obligation of the MS to adopt lists of prohibited actions. These prohibited actions included but were not limited to misleading or harassing practices, the collection of wrong or disputed amounts, debtor or third-party communications, and the bearing of debt collection costs (which could artificially and unjustifiably increase the debtor’s financial burden).

While the compromise text of the 2021 CSDP retained only a part of these amendments, they indicate that introducing substantive rules against abusive IDCPs is both possible and desirable if the EU wishes to create a functional internal market for NPLs while protecting consumers.

In my view, introducing substantive rules has several positive effects. *First,* it creates a level playing field for debt collectors and consumers by clarifying the supervision of abusive debt collectors. *Second,* it still allows MS to design their own rules to implement the substantive requirements, thus preserving local standards and traditions. In addition, MS have the opportunity to address issues left out by the EC to protect consumers and creditors. *Third,* having substantive and procedural rules serves as a valuable guide for other stakeholders (courts and supervising agencies). It would help clarify the case-by-case assessments of alleged breaches.

However, as in the case of the UCPD, a blacklist approach would be affected by inherent limitations. Its provisions would be too rigid and static, and rapid adjustments would not be possible (Stuyck et al., 2006). To address these flaws of the UCPD, legal scholars have suggested using the *Lamfalussy* approach toward regulation (Stuyck et al., 2006). While generally a sound proposal, this approach would be of little use regarding the regulation of abusive IDCPs absent a pan-EU body for consumer financial protection like the US Consumer Financial Protection Bureau (EESC, 2018, Sect. Procedural or Substantive? (Licenses v. Blacklists)). A more straightforward solution would be to implement standard and open-ended *de minimis* rules and enable MS to have national regulations (and regulators). These would ensure both flexibility and swiftness in addressing new or country-specific abusive IDCPs. The 2021 version of the CSDP would have achieved this had it not been reduced to a bare minimum by the need for political compromise.

### *Minimum or Maximum? (MS Autonomy vs. Market Efficiency*)

When implementing legislation seeking harmonization, the EU may choose between minimum and maximum harmonization (Craig & de Búrca, 2020). This choice determines whether MS can implement (or maintain) more stringent regulatory rules than those provided by the EU (Craig & de Búrca, 2020). Where maximum harmonization is sought, the corollary concern is whether consumer interests are adequately protected throughout the creation or strengthening of the internal market (Craig & de Búrca, 2020).

Abusive IDCPs are not exempt from such concerns. The best illustration stems from the wording of the UCPD, which, in the view of the EC, covers abusive debt collection (EC, 2016). Although the UCPD is a maximum harmonization instrument (UCPD, Recitals 14 and 15), it specifically allowed MS “to go beyond its provisions to protect the economic interests of consumers” given the “complexity and inherent serious risks” associated with financial services. In the EC’s view, these risks “necessitate detailed requirements, including positive obligations on traders” (UCPD, Recital 9 and Art 3(9)). There is no doubt that abusive IDCPs were perceived as part of the financial services that could be regulated more strictly at the MS level. Indeed, nine EU MS^5^ either maintained or adopted sector-specific regulation thereof^6^ following the implementation of the UCPD.

As legal scholars have noted, the total harmonization sought by the UCPD has clear advantages: cross-border market practices become more accessible, and consumers may benefit from better products and prices. However, it also has several drawbacks, as MS have no room to consider the particularities of their consumers and no opportunity for legislative experimentation (Stuyck et al., 2006).

From a policy-making perspective, I consider minimum harmonization preferable. On the one hand, it provides sufficient legal certainty to both consumers and debt collectors, ensuring that all EU consumers enjoy protection in the internal market. It is especially true for consumers in MS with inadequate or nonexistent sector-specific legislation. On the other hand, minimum harmonization also enables MS to address consumer threats specific to their national markets or have a broader impact on the national level provided they follow EU law (*Commission v Italy*, 2005). To date, no MS legislation concerning abusive IDCPs was found (or accused of being) in contradiction with EU legislation, even though the nine sector-specific laws have both commonalities and distinctive features.

Minimum harmonization is also the EC’s preferred choice for the regulation of credit servicers. The 2021 version of CSDP also advocates for the minimum approach by banning the most common abusive IDPCs at the EU level.

### Hard or Soft Law (Can One Trust Self-Regulation)?

Another relevant question regarding the regulation of abusive IDCPs in the EU is that of approach. Should the EU intervene and impose procedural and substantive rules (hard-law approach), such as in Belgium, Greece, Germany, Latvia, or Romania? Or should it allow debt collectors to adopt their own rules via internal policies or Codes of Conduct (soft-law approach), as in the Netherlands? Alternatively, should there be a mixture of both (hybrid approach), like in Denmark, Finland, and Sweden? As debt collection falls into the ambit of both the UCPD and the CSDP, the answer to this question will consider mainly these two instruments.

Initially, the CSDP took the hard-law approach only about procedural matters, such as licensing credit servicers (debt collectors and debt buyers). Policies for the fair treatment of consumer debtors were left to debt collectors and the MS. Although the 2021 version adds a minimum set of substantive standards of behavior (Council of the EU, 2021, Art 8a), the initial situation does not change significantly. The introduction of substantive rules applicable to abusive IDCPs does not entirely discard the EC’s apparent preference for a soft-law approach. Among the hard-law procedural requirements for licensing credit servicers, the CSDP states that servicers should have “an appropriate policy ensuring the fair and diligent treatment of the borrowers” (Council of the EU, 2021, Art 5 (1) d)) and present evidence thereof (Art 6 (2) f)) under the sanction of losing the authorization (Arts 7 (1) e) and 22 (1) c)). Thus, the EC seems satisfied with debt collectors’ internal standards of practice, an aspect underlined by the fact that none of the Recitals addresses the internal policies of the fair treatment of consumer debtors.

The EC’s silence is somewhat surprising when compared with other instances where the soft-law approach was encouraged. The EC also showed a propensity toward self-regulation via Codes of Conduct when it implemented the UCPD, even if Pavillon (Van Boom et al., 2014) points out that it did not grant any privileges to those who would voluntarily comply with a non-binding code. The UCPD (Recital 20) stated that in the legislator’s view, “the control exercised by the code owners at national or Community level to eliminate unfair commercial practices may avoid the need for recourse to administrative or judicial action and should therefore be encouraged.” To ensure a high level of consumer protection, the EC also recommended that consumer organizations are informed and involved in the drafting.

At the time, many legal scholars warned that self-regulation could not be relied upon. Stuyck et al. (2006) and Pavillon (Van Boom et al., 2014) claimed it could be “corporatist,” “can have potential anti-competitive effects,” and is “not always established in the general interest, but sometimes merely in the interest of the sector concerned.” Howells added that codes of conduct “only cover traders who sign up to them and are difficult to enforce” (Howells et al., 2006, Pavillon in Van Boom et al., 2014).

Others, however, focused on positive aspects, such as flexibility and the possibility to amend them to respond to market developments rapidly. They emphasized that self-regulation “could bring added value by implementing the principles of the directive in relevant sectors” and well-established codes of conduct “could reflect good business practice and be used to identify the requirements of professional diligence in concrete cases” (Abbamonte in Weatherill & Bernitz, 2007).

Several elements supported the soft-law approach of the UCPD. On the one hand was the willingness of other traders to follow the same code, coupled with the responsibility of an entity to monitor compliance. On the other hand, it is likely that once such a Code of Conduct has been firmly adopted, noncompliance with its provisions would constitute a misleading practice under Art 6 (2) and Annex I of the UCPD (Stuyck et al., 2006, Howells in Howells et al., 2006).

The CSDP leaves self-regulation to the whim of each trader and the MS. While the supervising authority could refuse to license traders whose policies are deemed inadequate, this opens the door to arbitrariness; absent clear standards, it would be hard to determine which approach is adequate or not. Assuming that such measures are adopted at the national level, they would revive fragmentation, which would impede the harmonization sought by the EC.

This position is in stark contrast with that of the MEPs. The advanced amendments to the CSDP would have turned the most common *de minimis* abusive practices into hard law across the union. At the same time, MS would still enjoy the freedom to implement additional rules at the national level or follow a soft-law approach. In my view, the MEPs’ proposals had merit, as notwithstanding the choice made by MS, the pan-EU ban of common abusive practices would have served better the harmonization efforts.

Finally, the language of the current hard-law provisions of the CSDP is a double-edged sword. On the one hand, the general wording would enable MS to update the list of practices and keep pace with the inventiveness of the industry. On the other, the language may appear vague, especially in the case-by-case assessment, thus making the task of judges or supervisors more difficult.

## Conclusions

The current concern of informal debt collection laws for the consumer’s privacy, psychological well-being, and protection from egregious practices stands in stark contrast with what was acceptable several decades ago or with the neoliberal concept of the self-reliant and fully responsible consumer. At the national level, where such protection against abusive IDCPs is available, debt collection legislation signals societal expectations about business behavior. Moreover, they represent a recognition that traditional remedies are not sufficient to empower the consumer to fend off abusive debt collection and establish trust in the integrity and proper functioning of the financial sector. Moreover, self-regulation and codes of conduct have proven unlikely to be effective (Brown, 2019). While oversight (via the authorization and selection of personnel) can help, consumers need a common set of behavioral standards for which there is accountability and clear individual redress in case of malpractice (Brown, 2019).

The question is why protection against abusive IDCPs is not equally available for all EU consumers. The explanation might be that establishing a single market for financial services and credit servicing is a neoliberal policy that rejects any regulation that might impede its smooth functioning. On the one hand, protecting all consumers from abusive IDCPs would deter the debt collection industry from engaging in cross-border activities. On the other hand, the harmonization of protection would send a strong signal that the EU is committed to pursuing a high level of consumer protection and rejects the idea of second-rate EU citizens.

The harmonization of regulation of IDCPs is a battle of both conflicting interests and political will. As the paper revealed, the EC pursued the neoliberal goal of fostering an internal market for credit servicing via the initial text of the CSDP, keeping its intervention to a minimum. Meanwhile, the European Parliament’s amendments aimed to bring its provisions in line with the welfare expectations of EU consumers by implementing a common set of standard rules of behavior. The 2021 version of the CSDP is a compromise in the true sense of the word.

In light of the previous analysis, I argue that the EU should have regulated abusive IDCPs via a dedicated instrument to cover all consumer-related debt collection without limiting itself to activities related to or arising from consumer credit. It could have done it by implementing a common set of open-ended, hard-law, *de minimis* rules and enabling EU MS to have national regulations (and regulators). These would have ensured both flexibility and swiftness in addressing new or country-specific abusive IDCPs. Notwithstanding the choices of the MS, a pan-EU ban of common abusive practices would have helped both harmonization efforts and would have ensured a high level of protection to consumers, especially in the 18 MS that have no sector-specific national rules in place. A sector-specific IDCP legislation would have provided cohesion and certainty all across the EU for all stakeholders beyond the ones offered by the 2021 version of the CSDP, assuming it will be adopted. Unfortunately, this solution would require significant political will and effort, which are unlikely to be achieved at this moment.

## Footnotes

1 The negative or absent answers from some of the respondents do not indicate that no abusive practices occurred in those MS, but that the respondent body did not receive complaints or was not aware of any.

2 The proponents highlight that there is a “clear EU dimension to reduce current stocks of NPLs, […] given the interconnectedness of the EU’s banking system” and the “important potential spill-over effects from Member States with high NPL levels to the EU economy as a whole.”.

3 The general nature of the UCPD results from Art 3.4, which it states that in case of conflict between its provisions and other EU rules regulating *specific aspects of unfair commercial practices* (emphasis added), the latter will prevail and apply to the specific aspects.

4 For instance: Denmark, Art 5 (6) and 8 (2) of Law 319/1997; Finland, Sect. 9 of Law no 411/2018; Belgium, Art 4(2) of Royal Decree of February 17, 2005; Germany, Art 12 (1) 1 a) of Legal Services Act of 12.12.2007; Italy, Art 11 of Consolidated Law on public security, approved by Royal Decree no 773/1931; and Latvia, Section III, 15.3 of Regulation no 64/2013.

5 The reference here is to the current MS of the EU. The UK also regulated abusive debt collection practices via sector-specific legislation when it was still a part of the EU.

6 The nine EU MS with sector-specific legislation dedicated to abusive debt collection practices are Belgium, Denmark, Finland, Germany, Greece, Latvia, the Netherlands, Romania, and Sweden (Stanescu 2021).
